# Supplementary material for: A personalized map of where, when, and how to stimulate the brain to elicit controlled responses
Source: bioRxiv. 2026 Jul 16:2026.07.16.738930. Preprint. [Version 1] doi: 10.64898/2026.07.16.738930 (PMC13405143; doi:10.64898/2026.07.16.738930)
Supplement: Supplementary file 1 [file NIHPP2026.07.16.738930v1-supplement-1.pdf]

bioRxiv preprint doi: https://doi.org/10.64898/2026.07.16.738930; this version posted July 16, 2026. The copyright holder for this preprint (which was not certified by peer review) is the author/funder, who has granted bioRxiv a license to display the preprint in perpetuity. It is made available under a CC-BY 4.0 International license.

Supplementary Materials

Supplementary Table S1 lists the symbols and response measures used in the perturbation analysis. The supplementary figures report the controls and robustness analyses referenced in the main text: the per-region task-activation reproduction maps for all seven tasks (Figure S1) and the modulation of the gating axis by cognitive state, with the gating and hierarchy persisting for task-driven states (Figure S2), and the functional-connectivity counterpart of the within-task decoding (Figure S3); subject specificity of the models (Figure S4), stability of the perturbation analysis across amplitudes (Figure S5), the neurotransmitter-receptor associations (Figure S6), the whole-brain versus local origin of the gating (Figure S7), the linear-model and sampling controls for the gating (Figures S8 and S9), and the cohort-level statistics of bifocal stimulation (Figure S10).

Table S1: **Symbols and response measures used in the perturbation analysis.** For each target region j, a virtual perturbation is applied at time t and the response is read at $t{+}1$; the quantities below summarize this response per region, per target, and over stimulation times. mean(GES), Var(GES) and CV(GES) are the response measures used in the main figures; the variance-explained measures are compared in Figure S7.

Symbol	Definition
Indices and states	
j	stimulus target region (ROI)
$x_i(t)$	baseline activity of region i at time t
$x_i(t{+}1)$	next unperturbed state of region i
$x_i^{(j)}(t{+}1)$	next perturbed (evoked) state of region i under stimulation at j
Energy measures	
$e_i(t) = x_i^2(t)$	local baseline energy
$E(t) = \sum_i x_i^2(t)$	global baseline energy (total ongoing activity)
$e_j^{(j)}(t{+}1) = \left(x_j^{(j)}(t{+}1)\right)^2$	local evoked energy at the target
$E^{(j)}(t{+}1) = \sum_i \left(x_i^{(j)}(t{+}1)\right)^2$	global evoked energy
Effect size (squared response)	
$\gamma_j^{(j)} = \left(x_j^{(j)} - x_j\right)^2$	local effect size at the target
$\Gamma^{(j)} = \sum_i \left(x_i^{(j)} - x_i\right)^2$	global effect size (GES), total squared response
Effect direction (signed response)	
$\delta_j^{(j)} = x_j^{(j)} - x_j$	local effect direction at the target
$\Delta^{(j)} = \sum_i \left(x_i^{(j)} - x_i\right)$	global effect direction
Per-target summaries (over stimulation times)	
$\langle \cdot \rangle_t$	time-average per target; mean(GES)= $\langle \Gamma^{(j)} \rangle_t$ is the responsiveness
$\mathrm{Var}_t(\cdot)$	temporal variance per target; Var(GES)= $\mathrm{Var}_t(\Gamma^{(j)})$ is the absolute variability
$\mathrm{CV}_t(\cdot)$	temporal coefficient of variation; CV(GES)= $\mathrm{SD}_t(\Gamma^{(j)})/\langle \Gamma^{(j)} \rangle_t$ is the relative variability
$\left[\mathrm{corr}(a,b)\right]^2$	variance in evoked b explained by baseline a (global E or local e_j; Fig. S7)

bioRxiv preprint doi: https://doi.org/10.64898/2026.07.16.738930; this version posted July 16, 2026. The copyright holder for this preprint (which was not certified by peer review) is the author/funder, who has granted bioRxiv a license to display the preprint in perpetuity. It is made available under a CC-BY 4.0 International license.

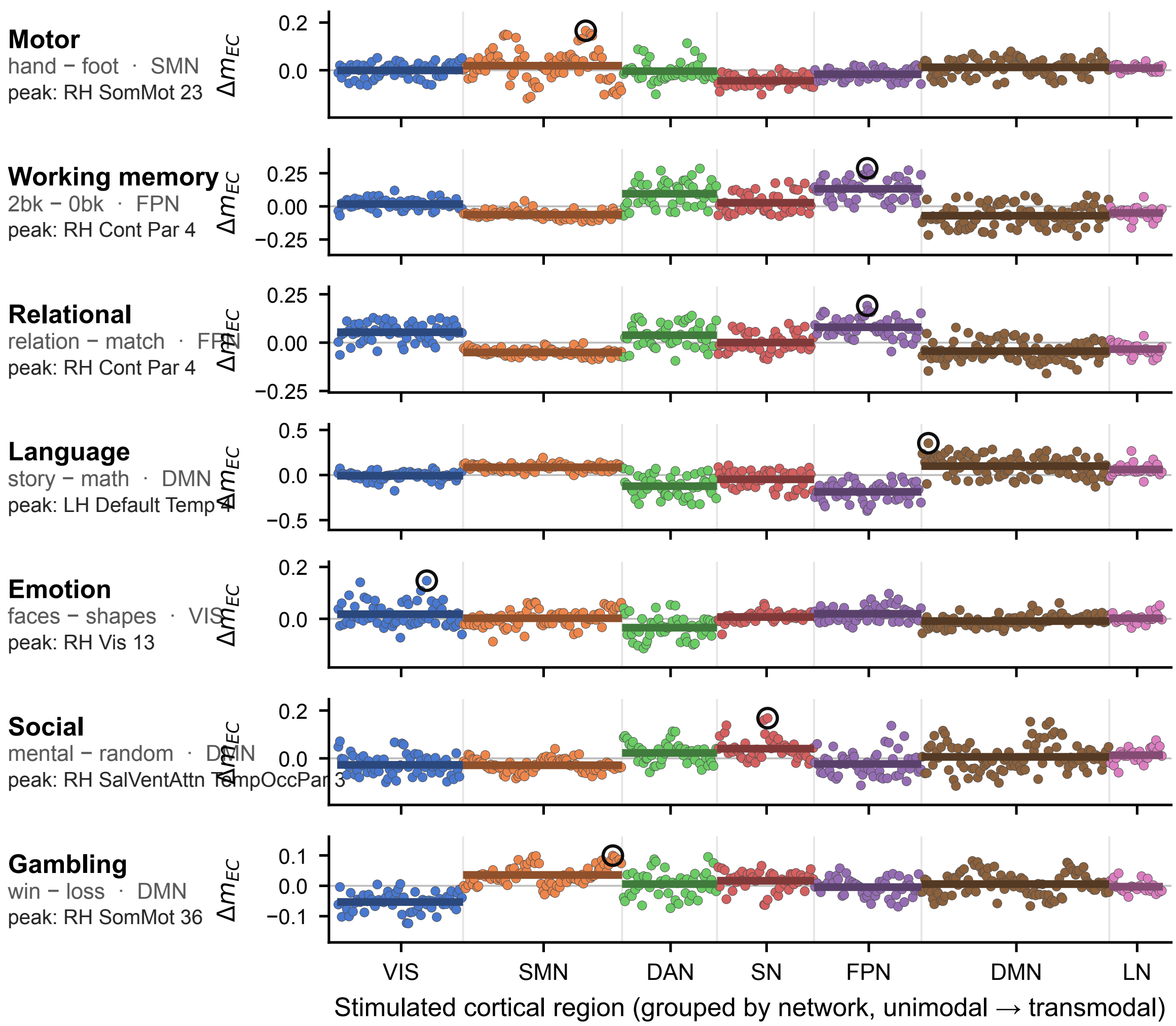

Figure S1: **Per-region maps of task-activation reproduction, all seven tasks.** For each task, the task-specific score Δm_{EC} (the evoked-map similarity of each region to the task activation, minus that region's mean across tasks) for every cortical region, grouped by Yeo-7 network (unimodal to transmodal) and colored by network. Dark horizontal lines are network means; the circled point is the peak region (labeled). The canonical network is elevated for the strong contrasts (motor, working memory, language, relational) and weaker for the subtler event-related contrasts (social, gambling).

bioRxiv preprint doi: https://doi.org/10.64898/2026.07.16.738930; this version posted July 16, 2026. The copyright holder for this preprint (which was not certified by peer review) is the author/funder, who has granted bioRxiv a license to display the preprint in perpetuity. It is made available under a CC-BY 4.0 International license.

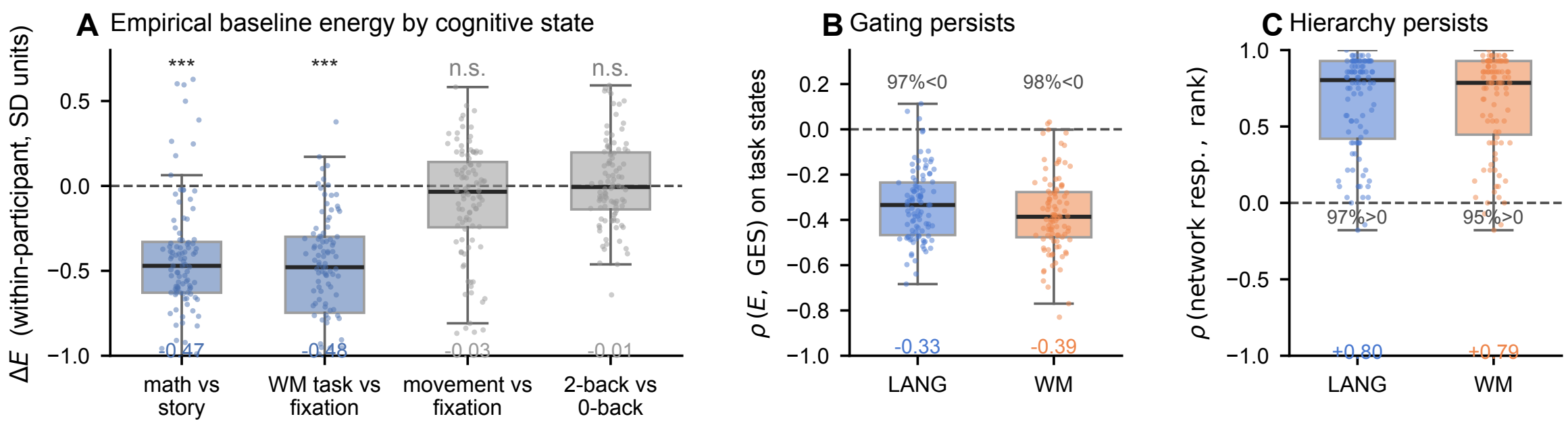

Figure S2: **Cognitive state modulates the gating axis. (A)** *Model-free.* Within-participant change in the empirical baseline energy $E(t) = \sum_i x_i(t)^2$ between cognitive states, in units of each participant's temporal standard deviation of E (box, cohort median and interquartile range; points, the 100 participants; dashed line, no change). Engagement and demand lower the baseline energy—arithmetic below narrative listening (math vs story, median −0.47 SD) and working-memory blocks below interior inter-block fixation (WM task vs fixation, −0.48 SD)—whereas brief movements (movement vs fixation) and parametric working-memory load (2-back vs 0-back) do not (medians printed below each box; $^{***}p < 10^{-3}$, n.s. $p > 0.05$, Wilcoxon signed-rank versus zero). Fixation is restricted to interior inter-block volumes to exclude run-edge non-steady-state states (Methods). Because the response grows as baseline energy falls, these lower-energy engaged states are the ones predicted to be more stimulable. **(B)** *The gating law generalizes to task-driven states.* Feeding each rest-trained model the task states and computing the global effect size (GES) at every cortical target, the per-participant Spearman correlation $\rho(E(t), \mathrm{GES})$ remains negative for both the language and working-memory tasks (medians −0.33 and −0.39; negative in 97% and 98% of participants), as at rest. **(C)** *The hierarchy persists.* The per-participant Spearman correlation between the seven network-mean responsiveness values and their unimodal-to-transmodal rank stays positive on task states (medians +0.80 and +0.79; positive in 97% and 95%), matching the resting hierarchy.

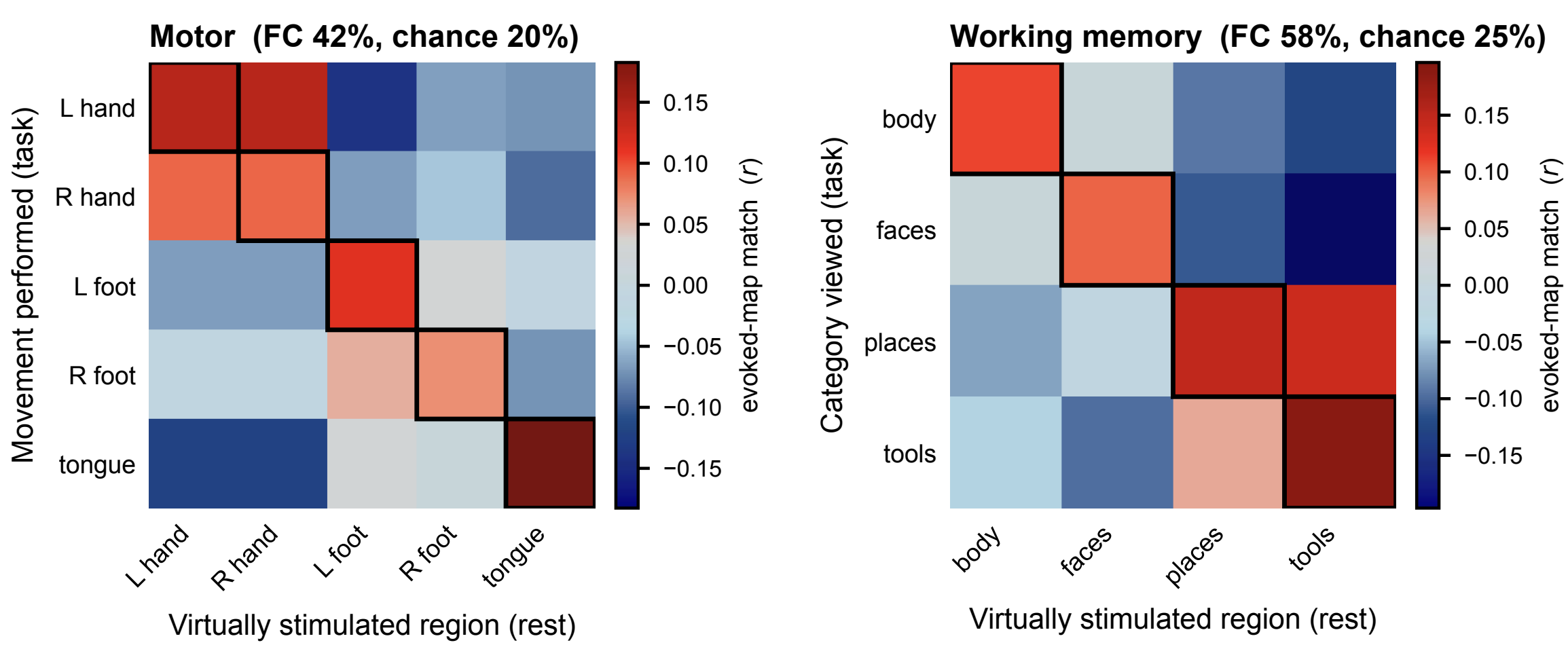

Figure S3: **Within-task decoding from functional connectivity (the FC counterpart of Figure 3C,D).** Confusion matrices for the motor (left) and working-memory (right) decoding when each condition's seed is the resting functional-connectivity map rather than the effective-connectivity map. Functional connectivity, lacking causal direction, smears the two hands and the two feet together (motor accuracy 42%, against 91% for effective connectivity) and separates the working-memory categories only coarsely (58% versus 68%). Rows, condition; columns, stimulated region; outlined diagonal, correct assignment.

bioRxiv preprint doi: https://doi.org/10.64898/2026.07.16.738930; this version posted July 16, 2026. The copyright holder for this preprint (which was not certified by peer review) is the author/funder, who has granted bioRxiv a license to display the preprint in perpetuity. It is made available under a CC-BY 4.0 International license.

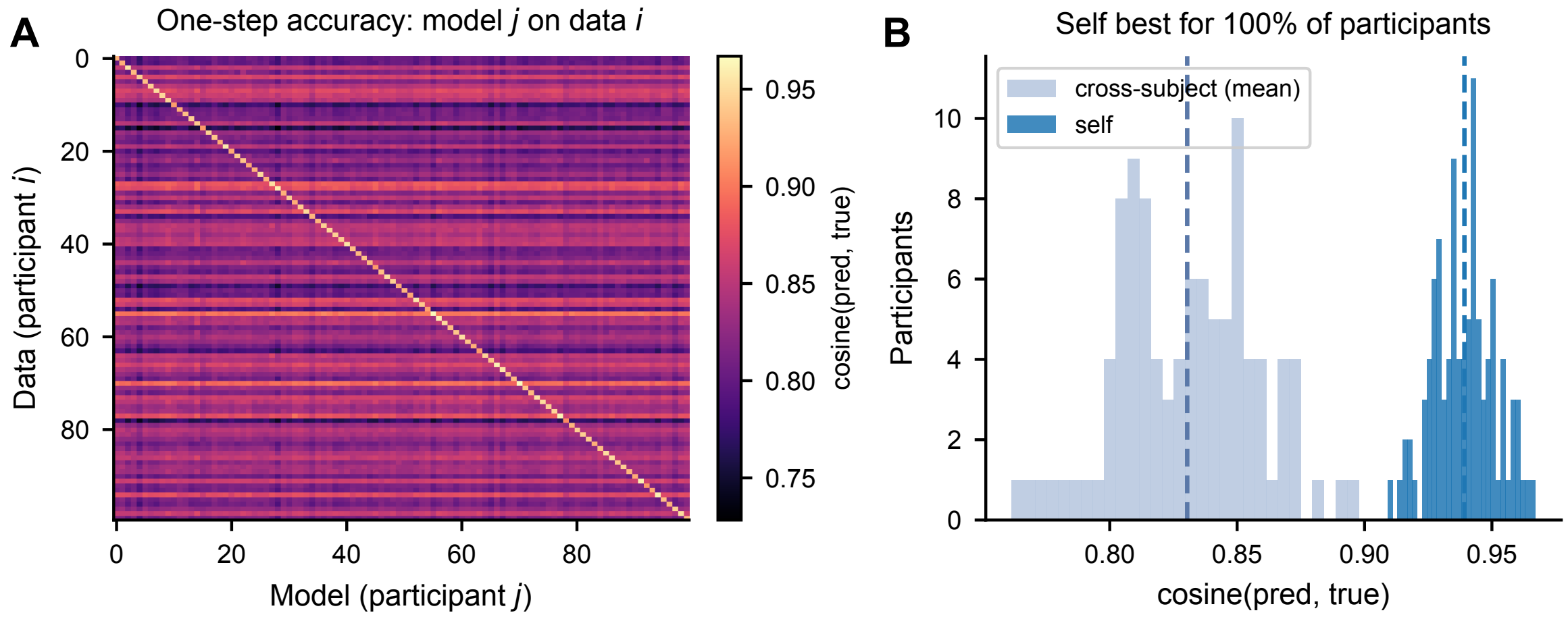

Figure S4: **The models are subject-specific: each predicts its own participant better than others.** For every participant we held out the last 20% of the recording and measured how well each of the 100 trained models predicts it one step ahead, scored by the cosine similarity between predicted and observed next-state vectors (scale-invariant). **(A)** Accuracy matrix: entry (i, j) is the accuracy of model j on participant i's held-out data. The bright diagonal shows that each model predicts its own participant best. **(B)** Distribution across participants of the self accuracy (model and data from the same participant) and the mean cross-subject accuracy. Self prediction is higher for every participant (median cosine 0.94 vs 0.83; the participant's own model is the single best-matching model in 100% of cases; Wilcoxon signed-rank $p < 10^{-17}$). The models capture individual dynamics rather than a generic template.

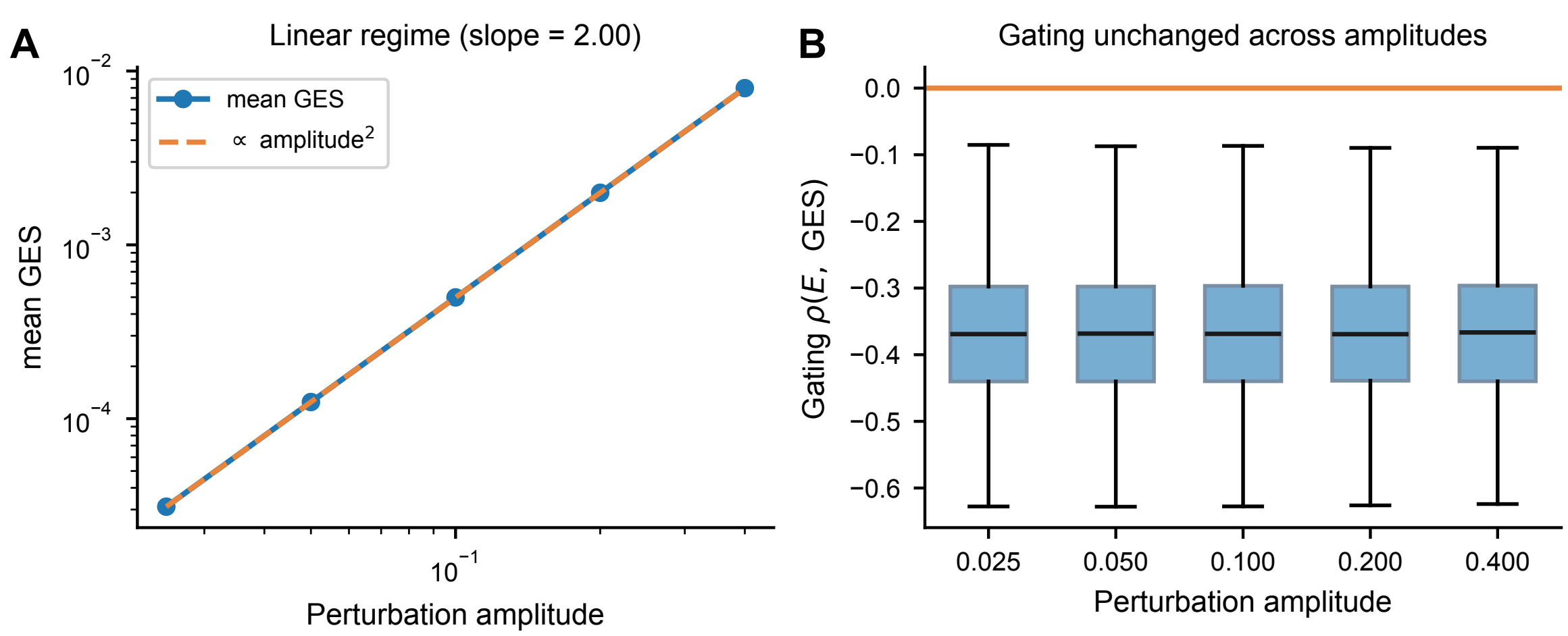

Figure S5: **The perturbation analysis is stable across stimulation amplitudes.** We repeated the virtual perturbation at amplitudes from 0.025 to 0.4 (in units of the standardized signal; the main analyses use 0.1) and recomputed the global effect size (GES). **(A)** The mean GES scales as the square of the perturbation amplitude (log–log slope = 2.00, dashed guide), confirming that the analysis operates in the linear-response regime where relative effects are amplitude-independent. The spatial map of responsiveness across cortical targets is essentially identical across amplitudes (Spearman $r = 1.00$ against the reference amplitude). **(B)** The state-gating is unchanged: the per-target correlation between baseline energy and GES is the same at every amplitude (median $\rho \approx -0.37$). Boxplots show the distribution over targets and participants.

bioRxiv preprint doi: https://doi.org/10.64898/2026.07.16.738930; this version posted July 16, 2026. The copyright holder for this preprint (which was not certified by peer review) is the author/funder, who has granted bioRxiv a license to display the preprint in perpetuity. It is made available under a CC-BY 4.0 International license.

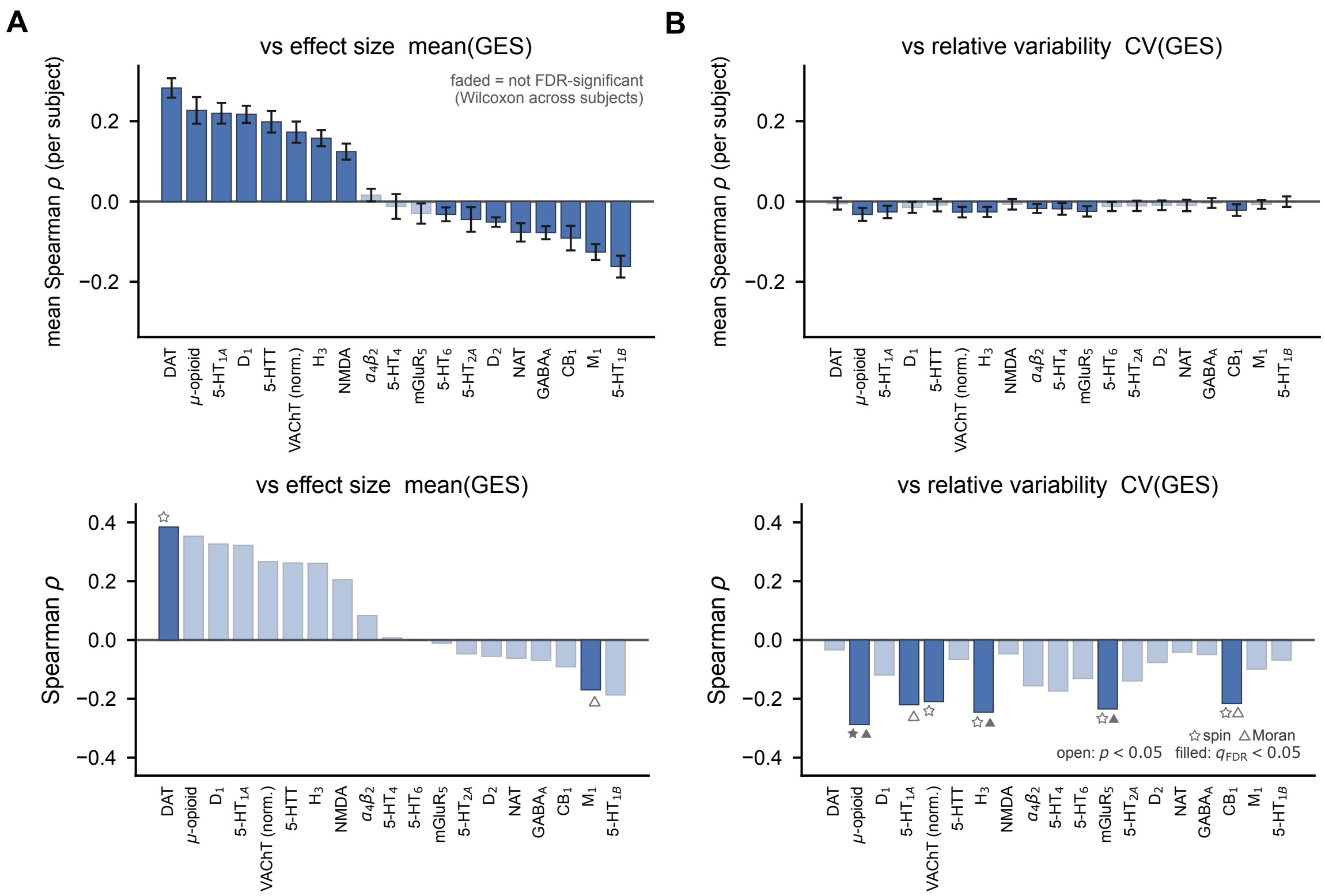

Figure S6: **Associations between the regional stimulation response and 19 neurotransmitter receptor and transporter densities.** Spearman correlations, across the 400 cortical regions, between each PET-derived density map and two properties of the response: its mean effect size, mean(GES) **(A)**, and its relative trial-to-trial variability, CV(GES) (standard deviation over mean of the effect size across stimulation times) **(B)**. Receptors are ordered by their correlation with mean(GES). *Top row, per-participant analysis:* for each participant, the receptor map is correlated with that participant's own regional map; bars show the mean correlation across the 100 participants (error bars, 95% confidence interval), and are faded when the across-participant distribution is not significant after FDR correction (Wilcoxon signed-rank versus zero). *Bottom row, group-level analysis:* the receptor map is correlated with the across-participant-mean regional map; significance is assessed against two spatial-autocorrelation-preserving nulls, the spin test (star) and Moran spectral randomization (triangle), with open markers indicating nominal $p < 0.05$ and filled markers survival of the Benjamini–Hochberg FDR correction across the 19 maps (Methods). Response magnitude has no FDR-robust molecular correlate (the dopamine transporter, DAT, is its strongest, nominal under the spin null only), whereas CV(GES) correlates negatively with several neuromodulators (μ-opioid, H_3 and $mGluR_5$ survive FDR under the Moran null), i.e. receptor-richer regions respond more reproducibly. These group-level CV associations do not reproduce per participant (top row).

bioRxiv preprint doi: https://doi.org/10.64898/2026.07.16.738930; this version posted July 16, 2026. The copyright holder for this preprint (which was not certified by peer review) is the author/funder, who has granted bioRxiv a license to display the preprint in perpetuity. It is made available under a CC-BY 4.0 International license.

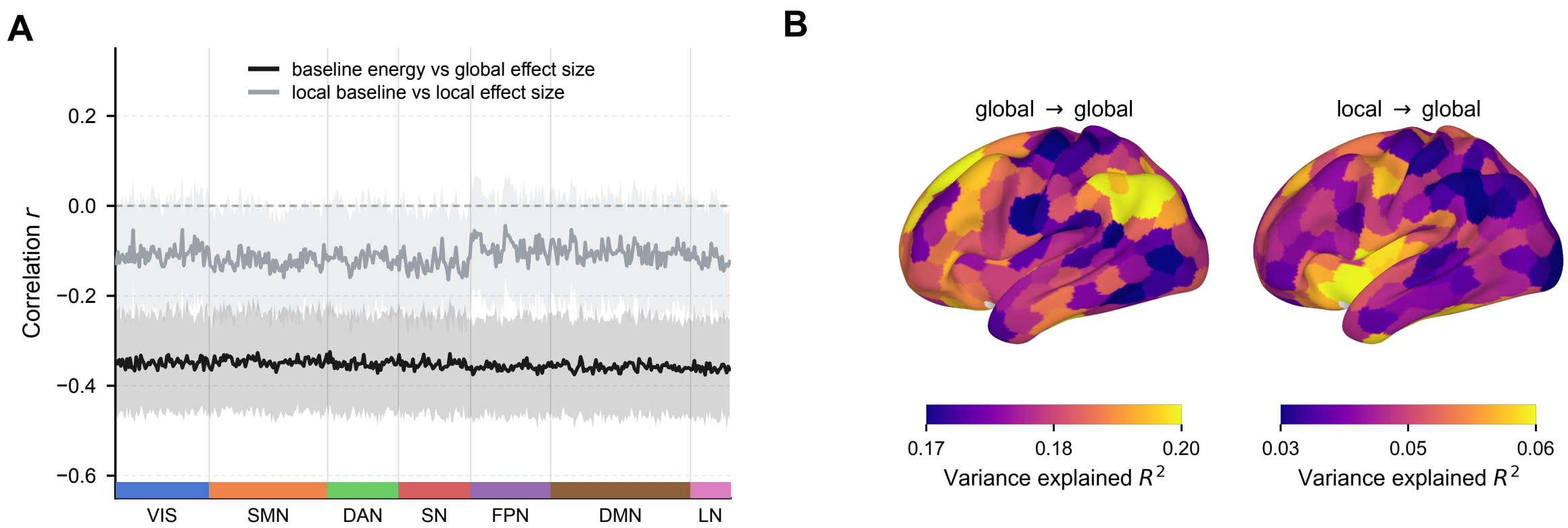

Figure S7: **The gating is driven by the whole-brain state, not the target's local activity. (A)** For each cortical region (ordered by Yeo-7 network), the across-time correlation between the brain's ongoing activity before stimulation and the resulting effect size, computed at two scales: the whole-brain baseline energy against the global effect size (black), and the target's local baseline activity against its local effect size (grey). Lines and shaded bands are the mean ± standard deviation across the 100 participants. The whole-brain correlation is consistently stronger (more negative) than the local one. **(B)** Variance in the global effect size explained (R^2) by the whole-brain baseline energy (global → global, left) and by the target's local baseline activity (local → global, right), shown on the cortical surface. The whole-brain state explains several-fold more variance than local activity at nearly every region.

bioRxiv preprint doi: https://doi.org/10.64898/2026.07.16.738930; this version posted July 16, 2026. The copyright holder for this preprint (which was not certified by peer review) is the author/funder, who has granted bioRxiv a license to display the preprint in perpetuity. It is made available under a CC-BY 4.0 International license.

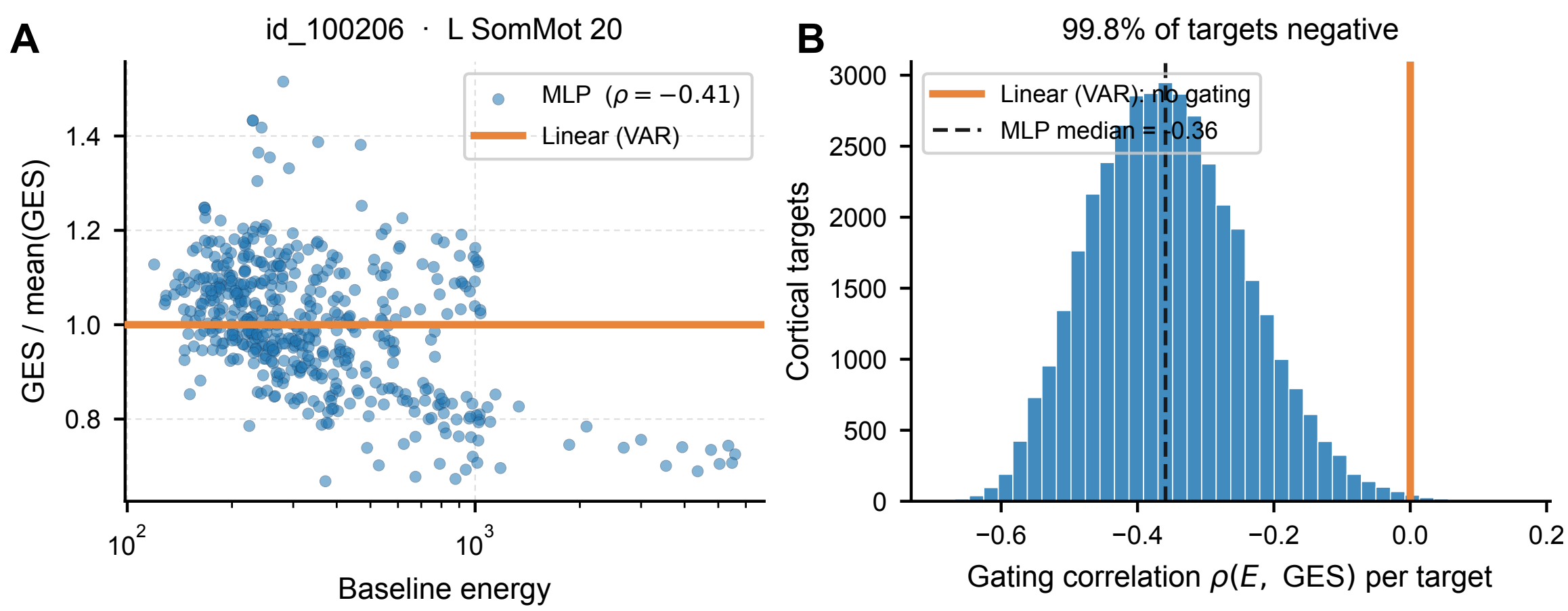

Figure S8: **The state-gating is a nonlinear property of the personalized model, absent in a linear model.** We fit an optimal linear one-step model (vector-autoregression, ordinary least squares, same $S = 3$ window) to each participant and ran the identical virtual-perturbation and global-effect-size (GES) computation used for the MLP model (Methods). For a linear map the evoked response is independent of the current state, so the GES is constant in time. **(A)** For the example participant and target of Figure 5A (id_100206, L SomMot 20), GES against baseline energy $E(t)$, normalized to each model's own temporal mean (the two models have different response magnitudes). The MLP response falls with baseline energy (Spearman $\rho = -0.41$); the linear model is constant (flat line). **(B)** Across cortical targets (100 participants, 40,000 targets), the distribution of the per-target gating correlation $\rho(E, \mathrm{GES})$ for the MLP (median -0.36, negative in 99.8% of targets). The linear model has zero temporal variance in the GES (coefficient of variation below 10^{-13} at every target and participant), so the correlation is undefined and the gating is absent by construction. The state-dependence therefore reflects nonlinear structure the model learns from the data, not the perturbation or normalization procedure.

bioRxiv preprint doi: https://doi.org/10.64898/2026.07.16.738930; this version posted July 16, 2026. The copyright holder for this preprint (which was not certified by peer review) is the author/funder, who has granted bioRxiv a license to display the preprint in perpetuity. It is made available under a CC-BY 4.0 International license.

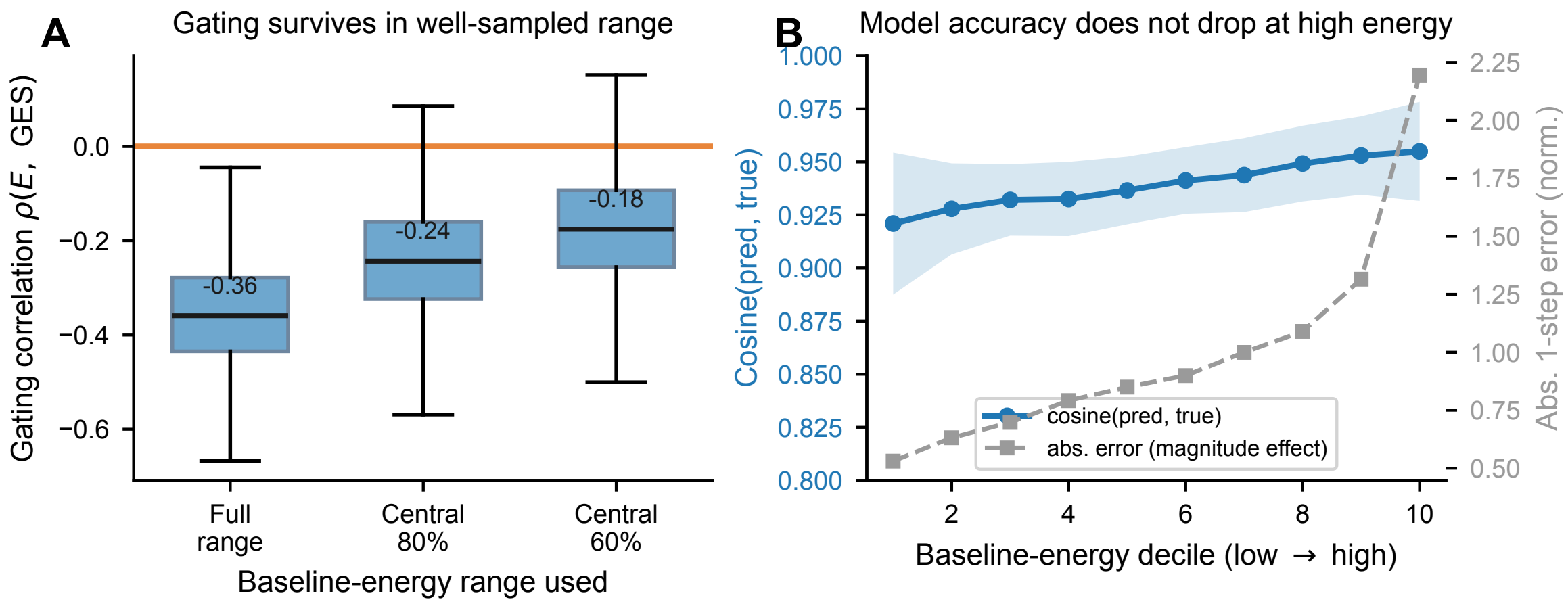

Figure S9: **The gating is not an artifact of sparse high-energy sampling.** High-energy states are less frequent than low-energy ones, so we checked that the gating is not produced by the model being poorly constrained where data are sparse. **(A)** Per cortical target (100 participants, 40,000 targets), the gating correlation $\rho(E, \mathrm{GES})$ computed over the full range of baseline energy, over the central 80% ([10,90] percentile), and over the central 60% ([20,80] percentile) of the energy distribution. The gating persists in the densely sampled middle (median $\rho = -0.18$, negative in 92.8% of targets over the central 60%); the attenuation is the expected consequence of restricting the predictor's range. The orange line marks $\rho = 0$. **(B)** One-step prediction accuracy of the model on held-out data, by baseline-energy decile (low to high). The scale-invariant cosine similarity between predicted and true next-state vectors (blue, left axis) stays high across the whole range and does not fall at high energy (0.92 to 0.96). The normalized absolute error (grey, right axis) rises with energy, but this reflects the larger signal magnitude of high-energy states rather than a loss of accuracy. The model is therefore well-fit across the energy range, so the smaller response at high energy is not a fitting artifact. Lines and bands are mean $\pm$ standard deviation across participants.

bioRxiv preprint doi: https://doi.org/10.64898/2026.07.16.738930; this version posted July 16, 2026. The copyright holder for this preprint (which was not certified by peer review) is the author/funder, who has granted bioRxiv a license to display the preprint in perpetuity. It is made available under a CC-BY 4.0 International license.

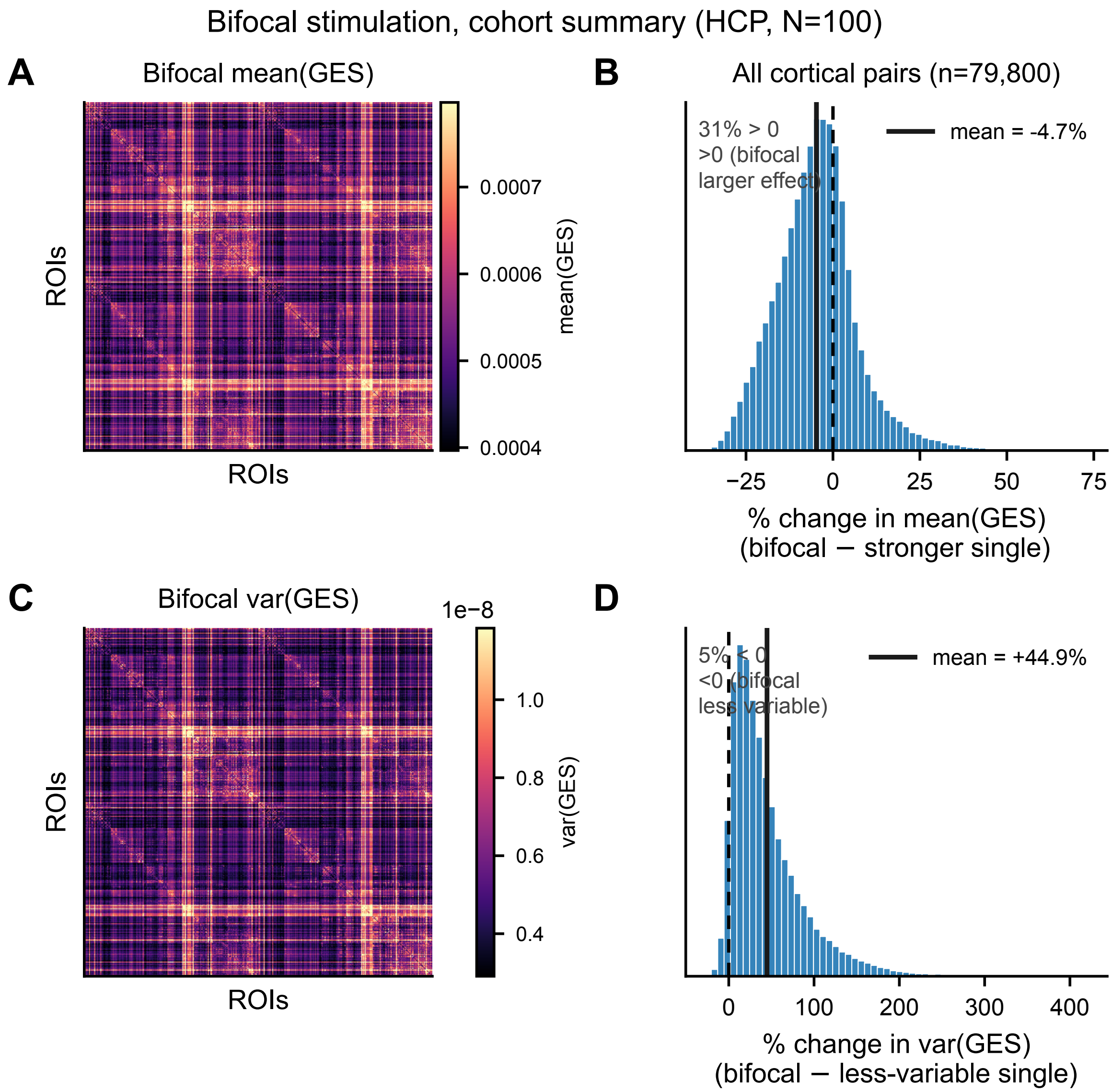

Figure S10: **Cohort statistics of bifocal stimulation across all cortical pairs (absolute scale).** Group-level summary of the bifocal analysis of Figure 6, over all 79,800 cortical region pairs, averaged across the 100 participants; the network-pair breakdowns are in Figure 6F,G. **(A)** Matrix of the bifocal mean effect size, mean(GES), for every region pair (rows and columns are cortical regions). **(B)** Distribution across all pairs of the percentage change in mean(GES) of bifocal co-stimulation relative to the stronger of the two single sites. Joint stimulation evokes a larger effect than the stronger single site for 31% of pairs (mean change −4.7%); the dashed line marks the mean. **(C)** Matrix of the bifocal absolute variance, var(GES). **(D)** Distribution of the percentage change in var(GES) relative to the less variable of the two single sites. Bifocal stimulation is less variable, in absolute terms, than the less variable single site for only 5% of pairs (mean change +44.9%): because the variance scales with the typically larger joint effect, co-stimulation usually raises the *absolute* variance even though it lowers the *relative* variability (CV) for most pairs (Figure 6C,E,G).
